# Medical Students as Nurses—II

**Published:** 1907-11-23

**Authors:** 


### MEDICAL STUDENTS AS NURSES.—II.

THE advance which trained nursing has made, from rough and ready ways to the skilled and finished manner in which the sick are tended in our best hospitals to-day, makes it essential for young doctors who are going forth to practise among the general public to acquaint themselves with the best methods of applying treatment so that they may be able when necessary to give practical instructions to those who are to carry out their orders. The question is how best to ensure that the necessary knowledge should be imparted to the senior students in a systematic manner. This problem seems to have been satisfactorily solved at the London Hospital, and for the last year or two we understand that demonstrations of medical and surgical nursing have been given in a systematic and yet unobtrusive manner, which has made these simple instructions acceptable to all concerned. The arrangement is that when a set of clinical clerks or a set of dressers are finishing their three months' term, during the last week a sister in charge of one of the wards of each physician and of each surgeon gives two demonstrations from 10 to 11 on Wednesday and Friday morning, at which the clinical clerks and dressers of the respective physicians and surgeons are expected by their house-physicians and house-surgeons to attend, the latter often setting an example by attending themselves. Due notice of these demonstrations and of the wards in which they will be held is given by the warden of the medical college, a few days before they take place, and a similar notice is put up on the students' notice-board in the hospital. Considerable care is taken over the preparation, so that time is saved by having all necessary appliances at hand. The matron's assistant goes through a rehearsal with each sister the previous week, to ensure that efficiency is maintained, and the probationers working in the respective wards get the benefit of these preparatory demonstrations. The demonstrations are given in ten wards on the same day, and at the same hour, and about six or eight students are expected at each demonstration. Occasionally there are a few more, who may have previously missed the opportunity, or who wish to attend a second time. The limited numbers enable all to see exactly what is being done, and to ask any questions they may find necessary. These demonstrations cannot be arranged in the summer term because of the holidays, but we learn that they take place with unfailing regularity at the end of October, the end of January, and the end of April.

We have no doubt that some similar plan will shortly be devised at other hospitals with medical schools attached. We append a syllabus of the details shown.

Any general definition of the proper distinction between the work of doctors and nurses and the essential difference in the education of each, to make both competent in their respective ways for their common work of helping the sick, is a good thing in itself, and it would be well if it were more generally recognised that each has something to learn from the other. Those responsible for the training of nurses would be well advised to make their probationers concentrate their efforts on endeavouring to perform practical nursing duties in a thoroughly competent way. It ought to be recognised that the welfare of patients depends on the united efforts of qualified doctors and efficient nurses, each bringing their own different and special knowledge to aid the patient's recovery, and to relieve suffering by every means that modern knowledge can bestow.

### DEMONSTRATIONS ON MEDICAL AND SURGICAL NURSING.—SYLLABUS:

#### MEDICAL.

1. How to change the under sheet and the draw sheet of a helpless patient.
2. How to lift helpless patients.
3. General care of patients' backs.
4. Arrangements to make patients comfortable. (Knee pillows, water pillows, water beds, hot-water tins, bed rests, etc., how to keep patients from slipping down in bed.)
5. How to make heart cases comfortable in bed.
6. The preparation and administration of enemata.
7. Care of patients' mouths.
8. Hot and cold sponging.
9. Hot-air bath and hot and cold pack.
10. Points in giving medicines, powders, and pills.
11. How to give hypodermic injections.
12. How to apply ice-bags, blisters, poultices, fomentations, leeches and dry [illegible]
13. Methods adopted to pancreatise and peptonise milk.
14. How to feed a troublesome infant.
15. How to keep a child still in bed.
16. How to keep heads clean and free from pediculi.

#### SURGICAL.

Many of the lectures on surgical nursing cover the same ground as above. The following additional subjects are introduced:—

1. The undressing of accident cases.
2. Treatment of patients with fractured spine.
3. Preparation of patients for serious operations.
4. Post anæsthetic vomiting.
5. Treatment of shock after operation (from a nursing point of view).
6. The feeding of patients after operation.
7. Arrangements for continuous transfusion.
8. The dressing of burns and the cleaning of tracheotomy tubes.
9. How to prevent children from soiling dressings.
10. Methods of counteracting shock in babies.

